# Patient-Reported Outcome Measures zur Erfassung der gesundheitsbezogenen Lebensqualität bei Patienten mit Stimm- und Schluckstörungen

**DOI:** 10.1007/s00106-023-01346-2

**Published:** 2023-08-07

**Authors:** Jörg E. Bohlender

**Affiliations:** 1 Abteilung Phoniatrie und Klinische Logopädie, Klinik für Ohren‑, Nasen‑, Hals- und Gesichtschirurgie, Universitätsspital Zürich, Universität Zürich, Frauenklinikstrasse 24, 8091 Zürich, Schweiz; 2 Universität Zürich, Zürich, Schweiz

**Keywords:** Erhebungen und Fragebögen, Klinische Relevanz, Qualität der medizinischen Versorgung, Gesundheitsstatus, Dysphagie, Surveys and questionnaires, Clinical relevance, Quality of health care, Health status, Dysphagia

## Abstract

Standardisierte und validierte Patientenbefragungen in Form von Fragebögen haben in der HNO-Heilkunde eine zunehmende Bedeutung. Die subjektive Bewertung von Symptomen und funktionellen Einschränkungen, aber auch der gesundheitsbezogenen Lebensqualität lassen sich mit sog. Patient-Reported Outcome Measures (PROM) erfragen. Diese Erhebungsinstrumente können neben der Anamnese und den objektiven Befunden als weitere wichtige Quelle zur Qualitätssicherung einer patientenzentrierten Versorgung genutzt werden. Im deutschsprachigen Raum gibt es mittlerweile einige PROM, die gezielt die Patientenperspektive bei Stimm- und Schluckstörungen erfragen. Bei Stimmpatienten werden v. a. die folgenden Fragebögen auf Basis des Voice Handicap Index (VHI), die auch international eingesetzt werden, angewandt: VHI-30, VHI-12i, VHI-9i. Im Bereich der oropharyngealen Dysphagie werden derzeit überwiegend die deutschen Versionen des Eating Assessment Tool-10 (EAT-10), des Sydney Swallow Questionnaire, SSQ (SSQ-G), des Swallowing Quality-of-Life Questionnaire (SWAL-QoL) und des MD Anderson Dysphagia Inventory (MDADI) eingesetzt.

## Abstract (en)

Standardized and validated patient surveys in the form of questionnaires are becoming increasingly important in otorhinolaryngology. Subjective symptoms and functional limitations but also health-related quality of life are assessed with so-called patient-reported outcome measures (PROMs). Besides patient history and objective findings, these questionnaires can be applied as another important source for ensuring the quality of patient-centered care. In German-speaking countries there are now several PROMs that specifically ask for the patient perspective on voice and swallowing disorders. For patients with voice disorders the following questionnaires based on the Voice Handicap Index (VHI), which are also used internationally, are mainly applied: VHI-30, VHI-12i, and VHI-9i. In the field of oropharyngeal dysphagia, the German versions of the Eating Assessment Tool-10 (EAT-10), des Sydney Swallow Questionnaire SSQ‑G, the Swallowing Quality of Life Questionnaire (SWAL-QoL), and the MD Anderson Dysphagia Inventory (MDADI) are currently mostly used.

Die individuelle Wahrnehmung und Bewertung einer Erkrankung sowie von spezifischen Krankheitssymptomen und ihrer Behandlung durch den Patienten werden als Patient-Reported Outcomes (PRO) bezeichnet. Mithilfe von Patient-Reported Outcome Measures (PROM) stehen Instrumente zur standardisierten Erfassung von PRO zur Verfügung. Im deutschsprachigen Raum gibt es eine Vielzahl von unterschiedlichen Fragebögen, welche es ermöglichen, die subjektive symptomspezifische Wahrnehmung bei Patienten mit Stimm- und Schluckstörungen zu dokumentieren und in das Behandlungsmanagement zu integrieren.

## Selbsteinschätzung von Stimm- und Schluckpatienten

Die Dokumentation der subjektiven Symptome und der gesundheitsbezogenen Lebensqualität ist ein wesentliches Kriterium in der Diagnostik und Therapieplanung von Patienten mit Stimm- und Schluckstörungen. Unabhängig von der klinischen Diagnostik wird der Schweregrad einer Stimm- oder Schluckstörung und die damit einhergehenden Einschränkungen trotz gleicher Erkrankung von Patient zu Patient unterschiedlich erlebt und bewertet. Diese individuelle patientenzentrierte Perspektive als eigenständiges Maß spiegelt sich im Konzept der subjektiven gesundheitsbezogenen Lebensqualität (Health Related Quality of Life, HRQoL) oder dem funktionellen Gesundheitsstatus („functional health status“) wider. Die HRQoL beschreibt im Idealfall 5 Dimensionen von Wohlbefinden und Funktionsfähigkeit (physisch, emotional, mental, sozial und verhaltensbezogen) aus Sicht der betroffenen Patienten. Im Gegensatz hierzu beschreibt der funktionelle Gesundheitsstatus den individuell erlebten Schweregrad eines spezifischen Symptoms, beispielsweise einer Schluck- oder Stimmstörung.

PROM verbessern die Kommunikation zwischen Patient und Behandler

Patient-Reported Outcome Measures (PROM) verbessern die Kommunikation zwischen Patient und Behandler. Die gesundheitsbezogene Lebensqualität und der funktionelle Gesundheitsstatus werden mithilfe von standardisierten Fragebögen (PROM) strukturiert und differenziert erfasst. Diese patientenzentrierte Sicht wird neben objektiven klinischen Daten als integraler Bestandteil eines zeitgemäßen klinischen Behandlungskonzepts verstanden. So lassen sich die Fragebögen zur Beurteilung von Behandlungsqualität und -kosten sowie zur Optimierung von Versorgungspfaden heranziehen. Letztlich sollen PROM die Kommunikation zwischen Patient und Arzt/Therapeut/Pflege verbessern helfen.

### Keine Erfassung ohne Konsequenzen

PROM optimieren die Identifizierung von individuellen krankheitsspezifischen Problemen, die nicht immer in der Anamnese zur Sprache kommen. Diese Aspekte sollten nicht nur mit dem Patienten angesprochen, sondern auch in das weitere klinische Behandlungsmanagement einbezogen werden. Somit sind PROM nur dann sinnvoll, wenn entsprechende Konsequenzen abgeleitet werden, da das alleinige Erfassen von PRO keine Verbesserung der Patientenversorgung garantiert. PROM sollten im Idealfall zu Beginn und Abschluss einer Therapie, aber auch im Intervall genutzt werden.

### Innovativer Ansatz: Digitalisierung von PROM

Die Digitalisierung von PROM findet derzeit im HNO-Bereich v. a. in hochspezialisierten Sprechstunden bei Patienten mit Kopf-Hals-Tumoren statt [21]. Im Rahmen der Tumornachsorge kann ein digitales innovatives PROM-Monitoring von komplexen objektiven und subjektiven Datensätzen erfolgen. Neben der Dokumentation von Stimm- und Schluckstörungen werden zusätzlich weitere bekannte Problembereiche von Tumorpatienten (u. a. Fatigue, Depression) mit passenden deutschsprachigen Fragebögen (u. a. Hospital Anxiety and Depression Score, HADS‑D; Health Questionnaire, PHQ‑D; Generalized Anxiety Disorder Questionnaire, GAD‑7; European Organization for Research and Treatment of Cancer Quality of Life of Cancer Patients, EORTC QLQ-C30; EORTC QLQ Head & Neck, EORTC QLQ H&N35) ergänzt und in ein onkologisches Gesamttherapiekonzept und Symptommanagement integriert.

## Stimmstörungen

Stimmstörungen können unabhängig von Alter, Geschlecht und sozialem Status auftreten. Die Ursachen sind vielfältig und lassen sich nicht immer auf ein rein organisches oder funktionelles Problem reduzieren. Funktionelle Auffälligkeiten können als Ursache oder Folge von strukturellen Befunden auftreten.

Stimmpatienten präsentieren sich nicht zwangsläufig mit einer hörbaren Heiserkeit

Zudem präsentieren sich Stimmpatienten nicht zwangsläufig mit einer hörbaren Heiserkeit. So kann eine Stimmerkrankung mit einer lediglich eingeschränkten stimmlichen Leistungsfähigkeit einhergehen und doch gleichzeitig die Lebensqualität des Betroffenen in Beruf und Freizeit massiv beeinträchtigen. Das übergeordnete Symptom „Dysphonie“ beschreibt daher alle denkbaren stimmbezogenen Beschwerden in den unterschiedlichsten Ausprägungsgraden. Eine moderne Stimmfunktionsdiagnostik ermöglicht die Objektivierung und Dokumentation von unterschiedlichsten Stimmbefunden. Die multivariate Stimmanalyse kann zur Beurteilung einer Therapieindikation beitragen und ermöglicht die prä- und posttherapeutische Evaluation stimmtherapeutischer und phonochirurgischer Maßnahmen. Seit 2001 besteht das Protokoll der European Laryngological Society (ELS) [7], welches zur differenzierten und standardisierten Beurteilung von Stimmerkrankungen neben der auditiv-perzeptiven Beurteilung, den instrumentellen akustischen Messungen, visuellen Schwingungsanalysen der Stimmlippen und aerodynamischen Messungen auch die Selbsteinschätzung des Patienten beinhaltet. Hierbei spielt der in unterschiedlichen deutschsprachigen Versionen vorliegende Voice Handicap Index (VHI) bei Stimmerkrankungen eine tragende Rolle. Der VHI kann bei symptomspezifischeren Phänomenen, die stimmassoziiert sein können (Reflux, Nackenbeschwerden usw.), ergänzt werden.

### Voice Handicap Index-30 (VHI-30)

International ist der in mehr als 30 Sprachen übersetzte Voice Handicap Index-30 (VHI-30) [10] in der klinischen Praxis sehr populär. Seit 2003 liegt der Fragebogen in einer deutschen [16] validierten Fassung vor. Der VHI-30 erfasst das Ausmaß der subjektiv erlebten stimmbezogenen Lebensqualität und besteht aus insgesamt 30 Fragen (Items) zu Problemen, die potenziell mit einer Stimmstörung einhergehen können. Der VHI-30 ist dabei in 3 Unterskalen mit je 10 Punkten unterteilt: physisch (P) bezieht sich auf die Wahrnehmung der Stimme des Patienten; emotional (E) gibt das emotionale Erleben des Patienten in Bezug auf seine Stimmprobleme wieder und funktional (F) bezieht sich auf die Kommunikationsprobleme des Patienten. In der Vergangenheit erfuhr der VHI-Fragebogen im Hinblick auf den erhöhten Zeitaufwand beim Beantworten der 30 Fragen eine vermehrte Kritik. Seit einigen Jahren stehen nun validierte gekürzte Versionen (Tab. 1) als Stimmstörungsindex (SSI = VHI12) und VHI-9i zur Evaluierung der stimmbezogenen Lebensqualität zur Verfügung.

**Table 1:** 

	Gesamtsumme*	Kein Handicap	Leichtgradiges Handicap	Mittelgradiges Handicap	Hochgradiges Handicap
VHI-30	0–120	0–14	15–28	29–50	51–120
VHI-12i	0–48	0–7	8–14	15–22	23–48
VHI-9i	0–36	0 ≤ 7	8 ≤ 16	17 ≤ 26	27 ≤ 36

Alle Versionen wenden für jede Frage (Item) als Antwortmöglichkeit die 5‑stufige Likert-Skala an: 0 (nie), 1 (selten), 2 (manchmal), 3 (oft) bis 4 (immer)

### Stimmstörungsindex

Der auf 12 Items reduzierte VHI deckt als kürzere Version des Originalfragebogens alle Dimensionen des ursprünglichen VHI-Fragebogens ab. Die abgespeckte Version stellt ebenfalls ein reliables und valides Messinstrument dar. Zunächst wurde die von Nawka entwickelte Kurzversion des VHI-12i als Stimmstörungsindex (SSI) benannt.

### VHI-9i

Eine weitere deutschsprachige validierte Kurzversion [5] ist der in 2009 publizierte internationale VHI-9i. Er umfasst nach einer erneuten Itemreduktion nur noch 9 Fragen. In der täglichen diagnostischen Praxis findet der VHI-9i im deutschsprachigen Raum eine häufige Verwendung.

### Vocal Tract Discomfort Scale

Im Vergleich zu den bekannten PROM VHI oder V‑RQOL, die den Fokus auf die stimmbezogene Funktion, Aktivität und Partizipation nach der Internationalen Klassifikation Funktionsfähigkeit, Behinderung und Gesundheit (ICF) richtet, steht mit der Vocal Tract Discomfort Scale (VTD Scale) ein zusätzliches Instrument zur Verfügung, das v. a. stimmbezogene Missempfindungen erfragt. Die deutschsprachige Vokaltrakt-Beschwerden-Skala (VTD-Skala) [15] beinhaltet 8 Symptome („Brennen“, „Enge“, „Trockenheit“, „Schmerzen“, „Kitzeln“, „Wund“, „Gereizt“, „Kloß im Hals“), die mit Stimmstörungen assoziiert sein können. Der Patient beurteilt zum einen die „Häufigkeit“ und zum anderen die „Intensität der Empfindung“ für jedes Symptom jeweils auf einer Skala von 0 (nie/keine) bis 6 (immer/extrem). Der Schweregrad der Beschwerden reicht von 0–13 (keine), 14–26 (leicht) und 27–40 (mittelgradig) bis zu 41–96 (hochgradig) [14].

### Vocal Fatigue Index

Ein weiteres stimmassoziiertes Phänomen wie „vocal fatigue“, im Deutschen als „pathologische Stimmermüdung“ bezeichnet, erfasst die Patientengruppe mit Stimmstörungen, die eine verminderte subjektive stimmliche Belastbarkeit mit zusätzlichen Hals- und/oder Nackenbeschwerden angeben. Die Erhebung der spezifischen Beschwerden von „vocal fatigue“ erfolgt seit 2015 mit dem in der englischen Sprache vorliegenden Vocal Fatigue Index (VFI). Eine deutschsprachige Übersetzung VFI‑D [20] wurde 2019 veröffentlicht. Der VFI umfasst 19 Fragen, die 3 Untergruppen zugeordnet werden: 1. Fragen zu stimmlichen Ermüdungs‑/Heiserkeitsempfindungen und Vermeidungsverhalten, 2. Fragen zu Beschwerden im Hals-Nacken-Bereich und 3. Fragen zur Verbesserung der subjektiven Symptomatik nach allgemeinem Ausruhen (sowohl Stimmruhe als auch körperliche und kognitive Entspannung). Insgesamt werden mit dem VFI nicht nur stimmbezogene Aspekte der pathologischen Stimmermüdung erfasst und bewertet.

### Singing Voice Handicap Index und SVHI-12

Im Gegensatz zum VHI, der primär subjektiv erlebte Einschränkungen der Sprechstimme abbildet, erhebt der 2007 veröffentlichte Singing Voice Handicap Index (SVHI) Beschwerden zur Singstimme. Dieser Fragebogen mit 36 Items, der in der deutschsprachigen Version vorliegt [13], dient nicht nur zur Selbstevaluation der physischen und emotionalen Auswirkungen einer gestörten Singstimme. Es werden im SVHI zudem soziodemografische Angaben (u. a. soziale und ökonomische Aspekte, Gesangsstil, Ausbildung) berücksichtigt.

Seit 2022 steht eine validierte deutschsprachige Kurzfassung mit 12 Fragen, SVHI-12, zur Verfügung

Seit 2022 steht nun eine validierte deutschsprachige Kurzfassung mit 12 Fragen, der SVHI-12 [8], zur Verfügung. Im SVHI und SVHI-12 werden anhand einer 5‑stufigen Likert-Skala Beschwerden erfragt. Zusätzlich wird die globale Einschätzung des Stimmproblems anhand einer 4‑stufigen Selbsteinschätzungsskala erhoben (0 – keine Störung, 1 – leichte Störung, 2 – mittelgradige Störung, 3 – hochgradige Störung). Diese gesondert erfragte globale Gesamteinschätzung des Patienten in Bezug auf die Singstimme stellt eine wichtige Bezugsgröße zu den einzelnen SVHI- und SVHI-12-Items dar. Aktuell wird der Gesamtscore von > 7 Punkten als gestörte Singstimme im SVHI-12 gewertet. Eine Differenzierung in eine leicht-, mittel- und hochgradige Singstimmstörung fehlt derzeit.

### Transsexual Voice Questionnaire for Male-to-Female Transsexuals

Mit dem Transsexual Voice Questionnaire for Male-to-Female Transsexuals (TVQ^MtF^) [18] steht seit 2020 eine Validierung der deutschsprachigen Übersetzung eines 30 Items umfassenden Stimmfragebogens vor, der systematisch und spezifisch die subjektive Sicht von trans*Frauen auf die eigene Stimme erhebt. Die 30 TWVQ-Fragen werden auf einer 4‑stufigen Likert-Skala beantwortet (1 = nie oder selten, 2 = gelegentlich, 3 = oft, 4 = meistens oder immer), die ermittelte Gesamtpunktzahl beträgt zwischen 30 und 120 Punkten. Die maximale Punktzahl von 120 Punkten bedeutet die größte Unzufriedenheit mit der eigenen Stimme in Relation zur individuellen Lebenssituation der betroffenen trans*Person. Gerade die Bewertung der stimmbezogenen Lebensqualität von trans*Frauen erlaubt aus der ärztlichen und therapeutischen Perspektive ein besseres Verständnis der individuellen Bedürfnisse und die Selbstwahrnehmung der Stimme. Der TVQ^MtF^ als PROM ergänzt die spezifische Anamnese und die klinische Untersuchung und misst v. a. die subjektive Bewertung vor und nach einer logopädischen Stimmtherapie oder einer stimmfeminisierenden Operation.

### Fragebogen zur Erfassung des stimmlichen Selbstkonzepts

Der 2015 auf Deutsch publizierte Fragebogen zur Erfassung des stimmlichen Selbstkonzepts (FESS) [17] besteht aus 17 Items, die auf einer 5‑stufigen Likert-Skala (1–5) beantwortet werden. Dabei wird das persönliche Erleben der eigenen Stimme in 3 Einzelskalen erfasst: (1) Beziehung zur eigenen Stimme (6 Items), (2) Bewusstheit im Umgang mit der Stimme (6 Items) und (3) Zusammenhang zwischen Stimme und Emotion (5 Items). Für die ersten beiden Einzelskalen kann jeweils ein Gesamtpunktwert von 5–30, für die dritte Einzelskala ein Gesamtpunktwert von 5–25 erreicht werden.

Im Unterschied zu symptomorientierten PROM wird im FESS die persönliche Beziehung zur Stimme bewertet

Im Unterschied zu den üblichen symptomorientierten PROM (u. a. VHI) wird im FESS die persönliche Beziehung zur Stimme bewertet. Der FESS stellt insbesondere die stimmlichen individuellen Beziehungs- und Erlebensaspekte hinsichtlich der eigenen Stimme in den Mittelpunkt der Untersuchung.

### Fragebogen zur stimmbezogenen Lebensqualität

Der Fragebogen zur stimmbezogenen Lebensqualität als die deutschsprachige und validierte Version des Voice-Related Quality of Life [9, 19], V‑RQOL, beinhaltet 10 Fragen zur Beurteilung der stimmbezogenen Lebensqualität. Die 10 Items des V‑RQOL beinhalten sozial-emotionale (4 Items) und physisch-funktionelle (6 Items) Aspekte. Die Antwortmöglichkeiten reichen von 1 (kein Problem), 2 (kaum ein Problem), 3 (schon ein Problem), 4 (ein großes Problem) bis 5 (ein Problem, wie es schlimmer nicht sein könnte). V‑RQOL-Werte von 0–40 werden als „klinisch auffällig“ gewertet, eine Punktzahl von 41–80 entspricht „keiner klinisch bedeutsamen Einschränkung“, und Werte über 80 bedeuten eine „normale, unbeeinträchtigte stimmbezogene Lebensqualität“. Der V‑RQOL wurde mit seinen 10 Items als eine geeignete zeitsparende Option zu dem ursprünglich 30 Fragen umfassenden VHI entwickelt. Mit dem VHI-12i und VHI-9i stehen jedoch mittlerweile Kurzfassungen der Originalversion als Alternativen zur Verfügung, weshalb der V‑RQOL nicht die breite Anwendung in der klinischen Praxis erfahren hat.

### Mit Stimmstörungen assoziierte PROM

#### Neck Disability Index (NDI-G)

Die deutsche Version des Neck Disability Index (NDI-G) [6] ist ein 10 Items umfassender Selbstbeurteilungsfragebogen zu Beeinträchtigungen durch Nackenprobleme im Alltag. Der NDI‑G dient der Erfassung von Beschwerden und Problemen bei alltäglichen Aktivitäten, die durch die Halswirbelsäule verursacht werden können.

Nackenbeschwerden werden z. T. bei Stimmpatienten mit vermehrter Halsmuskelanspannung angegeben

Das diffuse Symptombild Nackenbeschwerden wird teilweise bei einer Gruppe von Stimmpatienten angegeben, die bei der Stimmbildung über eine vermehrte Muskelanspannung im Bereich des Halses klagt. So wird diese vermehrte und umschriebene muskuläre Verspannung direkt im Kehlkopfbereich, paralaryngeal, im Bereich des Nackens, Schultergürtels, Rachens oder der Zungenregion selbst angegebenen. Als Ursachen für diesen muskulären Hypertonus und einer begleitenden Stimmstörung, im angloamerikanischen als „muscle tension dysphonia“ (MTD) definiert, werden eine inadäquate Stimmtechnik, ein Mangel an stimmlicher Ausbildung, ungünstige Arbeitsbedingungen für stimmintensive Tätigkeiten, die Persönlichkeitsstruktur und psychische Störungen diskutiert [12]. Die 10 Items werden jeweils mit 0 bei geringen Beschwerden und mit maximal 5 Punkten bei stärksten Beschwerden bewertet. Die Maximalpunktzahl beträgt 50 Punkte. Die erreichte Punktzahl wird zunächst durch die mögliche Gesamtpunktzahl 50 geteilt. Dieser Wert wird mit 100 % multipliziert und ergibt den Score im ND. Ein Gesamtwert von ≤ 8 kennzeichnet Patienten mit einer geringen Symptomatik, hingegen bedeutet der Gesamtscore > 40 ein sehr ausgeprägtes Beschwerdebild.

#### Reflux Symptom Score-12 Questionnaire

Mit dem ins Deutsche übersetzten Reflux Symptom Score 12 Questionnaire (G-RSS-12) [4] liegt ein Werkzeug zur Erfassung der Häufigkeit und Schwere der durch laryngopharyngealen Reflux (LPR) bedingten Symptomatik und der damit einhergehenden Einschränkung der Lebensqualität vor. Insgesamt präsentiert sich der LPR daher durch ein sehr breites Spektrum an teils sehr unspezifischen Symptomen. Diese können u. a. Heiserkeit bis hin zu einem hyperreagiblen Larynx umfassen. Die Häufigkeit und der Schwergrad der Symptomatik werden mit einer 6 Punkte umfassenden Likert-Skala evaluiert. Für jedes einzelne Item werden die Wertungen für Häufigkeit und Schweregrad der Symptomatik multipliziert und anschließend miteinander addiert. Daraus ergibt sich der RSS-12-Score. Ergebnisse über 11 werden als pathologisch gewertet. Dabei gilt bzgl. Häufigkeit („frequency“): 0: keine Beschwerden im letzten Monat. Für 1, 2, 3 und 4 gilt entsprechend: 1–2; 2–3, 3–4 und 4–5 × Beschwerden pro Woche im letzten Monat. 5: die Beschwerden bestehen täglich. Für Schwere („severity“) und Beeinträchtigung der Lebensqualität („quality of life impact“) gilt: 0 (kein Problem) bis 5 (schweres Problem).

## Dysphagie

Patienten mit einer Schluckstörung im Kopf-Hals-Bereich weisen vielfältige Symptome auf, welche von einem zervikalen Globusgefühl bis hin zu einer objektivierbaren schwerwiegenden Schluckunfähigkeit reichen können. Die zugrunde liegenden Ursachen sind vielfältig und erklären dabei häufig nur teilweise die Art und den Grad der Funktionseinbußen beim Schlucken. Der Schweregrad einer Schluckstörung und die damit einhergehenden subjektiven Einschränkungen werden trotz gleicher Erkrankung unabhängig von der klinischen Diagnostik von Patient zu Patient unterschiedlich erlebt und bewertet. Im deutschsprachigen Raum stehen derzeit 4 validierte dysphagiespezifische Fragebögen mit unterschiedlichen psychometrischen Eigenschaften zur Verfügung: SWAL-QOL [11], MD Anderson Dysphagia Inventory (MDADI) [1], Eating Assessment Tool-10 (EAT-10) und die deutsche Version des Sydney Swallow Questionnaire SSQ (SSQ-G).

### Eating Assessment Tool-10

Der deutschsprachige G‑EAT-10 erfüllt die Kriterien eines funktionellen Gesundheitsstatus-Fragenbogens [22]. Er besteht aus insgesamt 10 Fragen, die spezifische Symptome, aber auch emotionale, soziale und psychologische Aspekte einer Schluckstörung erfassen. Eine 5‑Punkte-Likert-Skala erfasst Antwortmöglichkeiten zwischen 0 und 4, dabei entspricht 0 „kein Problem“, 4 „schwerwiegendes Problem“. Die maximal ermittelbare Gesamtpunktzahl beträgt 40 Punkte.

Vorteile des EAT-10 sind die gute Lesbarkeit und der geringe Zeitaufwand beim Ausfüllen

Ein Cut-off-Wert wurde in der Originalpublikation von Belafsky et al. [2] schon mit 3 Punkten bestimmt. Vorteile des in mehreren Sprachen validierten EAT-10 zu anderen PROM sind die gute Lesbarkeit und der geringe Zeitaufwand beim Ausfüllen des Fragebogens durch den Patienten.

### Sydney Swallow Questionnaire

In 17 Fragen erhebt der deutschsprachige G‑SSQ [3] subjektive Schluckschwierigkeiten in Bezug auf die Konsistenz des Bolus, Einschränkungen in der oralen und pharyngealen Phase einschließlich des velopharyngealen Abschlusses sowie Anzeichen einer Penetration, Aspiration oder nasalen Regurgitation. Die Fragen lassen sich dabei den 3 Unterskalen global, physisch bzw. schluckbezogene Lebensqualität zuordnen. Der Patient beantwortet mit einem Kreuz auf einer 100 mm langen visuellen Analogskala (VAS) die jeweilige Frage. Ein Kreuz auf der linken Seite bei 0 mm entspricht der Merkmalsausprägung „nicht vorhanden/ausgeprägt“ und rechtsseitig bei 100 mm „maximal ausgeprägt“. Einzige Ausnahme ist Frage Nr. 12 „Wie lange benötigen Sie für eine Mahlzeit?“ mit einer numerischen Auswertungsskala von 0–5. Zur Auswertung wird die Antwort auf Frage Nr. 12 mit 20 multipliziert. Es kann ein maximales Gesamtergebnis von 1700 Punkten (maximal ausgeprägte subjektive Schluckstörung) erreicht werden [3].

### Swallowing Quality of Life Questionnaire

Der 44 Items umfassende deutschsprachige SWAL-QOL [11] beinhaltet 10 Unterskalen: Anstrengung, Verlangen nach Essen, Zeitdauer zum Essen, Essensbeschränkung, Kommunikation, Angst, Gesundheit, Alltagsfunktion, Schlaf, Ermüdung. Die Antwortmöglichkeiten werden mit einer 5‑stufigen Likert-Skala erfragt. Außerdem wird gesondert die Symptomhäufigkeit erhoben und bewertet. Niedrig ermittelte Prozentwerte werden einer herabgesetzten schluckbezogenen Lebensqualität zugeordnet. Eine differenzierte Graduierung des SWAL-QOL fehlt.

### MD Anderson Dysphagia Inventory

Die deutsche Validierung des MDADI [1] erfolgte an 102 Patienten mit einem Karzinom der Mundhöhle. Er beinhaltet 20 Fragen, welche sich globalen, emotionalen, funktionellen und physischen Unterskalen zuordnen lassen. Die Fragen werden auf einer 5‑stufigen Likert-Skala („stimme voll und ganz zu“ bis hin zu „stimme auf keinen Fall zu“) beantwortet. Die komplexe Berechnung des MDADI-Gesamtscores erfolgt aus den Unterskalen emotional, funktional und physisch. Die Unterskala „global“ wird gesondert ausgewertet. Wie beim SWAL-QOL wird ein niedriger MDADI-Gesamtscore mit einer geringen schluckbezogenen Lebensqualität in Verbindung gebracht.

## Fazit für die Praxis

- Es existiert eine Vielzahl an validierten deutschsprachigen Patient-Reported-Outcome-Measures (PROM) bei Stimm- und Schluckpatienten.
- PROM erfassen symptomspezifische Aspekte aus der Sicht der betroffenen Patienten.
- PROM sollten nur eingesetzt werden, wenn Konsequenzen für die Versorgung der Patienten abgeleitet werden.
- Eine Kombination aus verschiedenen PROM bildet eine differenziertere Patientenperspektive ab.
